# Neurologic Correlates of Gait Abnormalities in Cerebral Palsy: Implications for Treatment

**DOI:** 10.3389/fnhum.2017.00103

**Published:** 2017-03-17

**Authors:** Joanne Zhou, Erin E. Butler, Jessica Rose

**Affiliations:** ^1^Department of Orthopaedic Surgery, Stanford UniversityStanford, CA, USA; ^2^Motion and Gait Analysis Lab, Lucile Packard Children’s HospitalPalo Alto, CA, USA; ^3^Thayer School of Engineering, Dartmouth CollegeHanover, NH, USA; ^4^Neukom Institute for Computational Sciences, Dartmouth CollegeHanover, NH, USA

**Keywords:** cerebral palsy, brain injury, neuroimaging, neuromuscular deficits, gait

## Abstract

Cerebral palsy (CP) is the most common movement disorder in children. A diagnosis of CP is often made based on abnormal muscle tone or posture, a delay in reaching motor milestones, or the presence of gait abnormalities in young children. Neuroimaging of high-risk neonates and of children diagnosed with CP have identified patterns of neurologic injury associated with CP, however, the neural underpinnings of common gait abnormalities remain largely uncharacterized. Here, we review the nature of the brain injury in CP, as well as the neuromuscular deficits and subsequent gait abnormalities common among children with CP. We first discuss brain injury in terms of mechanism, pattern, and time of injury during the prenatal, perinatal, or postnatal period in preterm and term-born children. Second, we outline neuromuscular deficits of CP with a focus on spastic CP, characterized by muscle weakness, shortened muscle-tendon unit, spasticity, and impaired selective motor control, on both a microscopic and functional level. Third, we examine the influence of neuromuscular deficits on gait abnormalities in CP, while considering emerging information on neural correlates of gait abnormalities and the implications for strategic treatment. This review of the neural basis of gait abnormalities in CP discusses what is known about links between the location and extent of brain injury and the type and severity of CP, in relation to the associated neuromuscular deficits, and subsequent gait abnormalities. Targeted treatment opportunities are identified that may improve functional outcomes for children with CP. By providing this context on the neural basis of gait abnormalities in CP, we hope to highlight areas of further research that can reduce the long-term, debilitating effects of CP.

## Introduction

Cerebral palsy is the most common movement disorder in children, with an overall prevalence worldwide of 2–3 per 1,000 live births, and a much higher prevalence of 60–150 per 1,000 among neonatal survivors weighing less than 1500 grams at birth (Oskoui et al., 2013). A diagnosis of CP is often made based on the observation of abnormal muscle tone or posture, delayed motor milestones, or the presence of gait abnormalities in young children, which range from mild, i.e., toe-walking, to severe, i.e., crouched, internally rotated gait (Wu et al., 2004). Gait begins to stabilize around age 3–4 years and matures by 7 years of age (Wu et al., 2015). Among children with CP who are not walking by age 2 years, only 10% walk independently by age 7 (Wu et al., 2004), underscoring the importance of early identification and intervention. In recent years, 3D gait analysis has become the gold standard for delineating gait abnormalities in children with CP (Gage and Novacheck, 2001). Furthermore, neuroimaging of high-risk neonates and of children diagnosed with CP have identified patterns of neurologic injury associated with CP. However, the link between neurologic injury, neuromuscular deficits, and specific gait abnormalities in CP is not well understood. This review of the neural basis of gait abnormalities in CP discusses what is known about links between the location and extent of brain injury and the type and severity of CP, in relation to the associated neuromuscular deficits, and subsequent gait abnormalities. We discuss current literature that addresses the nature of the brain injury in CP, as well as the neuromuscular deficits and subsequent gait abnormalities in CP on both a microscopic and functional level. Ultimately, we hope that this review clarifies some of the neurologic correlates of gait abnormalities and points to areas of further research that can improve functional outcomes for children with CP.

## Brain Injury in Cerebral Palsy

Cerebral palsy describes “a group of permanent disorders affecting the development of movement and posture, causing activity limitation, that are attributed to non-progressive disturbances that occurred in the developing fetal or infant brain” (Rosenbaum et al., 2007). Although the initial brain injury is non-progressive, the musculoskeletal impairments and functional limitations associated with CP are indeed progressive (Bell et al., 2002; Sanger, 2015). A loss of oxygen to the developing brain, i.e., hypoxia-ischemia, is the primary mechanism by which brain injury occurs in CP. Other causes of brain injury include hemorrhage, infection, metabolic derangement, brain malformation, and bilirubin neurotoxicity. In contrast, birth asphyxia resulting in CP is relatively rare, accounting for <10% cases, (Paneth et al., 2006; Strijbis et al., 2006; Ellenberg and Nelson, 2013), and evidence of genetic influences on development of CP is newly emerging and thought to contribute to up to 30% of CP cases (Costeff, 2004; Moreno-De-Luca et al., 2012; MacLennan et al., 2015). The prevalence of CP increases with prematurity, ranging from 1.1 per 1000 infants born at 40 weeks’ gestation to 90.7 per 1000 infants born at 26 weeks’ gestation (Trønnes et al., 2014). The risk of CP decreases linearly with increasing gestational age: 8.5% for 23–27 weeks, 5.6% for 28–30 weeks, 2.0% for 31–33 weeks, 0.4% for 34–36 weeks, and 0.2% for 37+ weeks gestation. Male sex is also a general risk factor, as the rate of CP per 1000 male births exceeds that among females by about 30% (Jarvis et al., 2005).

**FIGURE 1:**
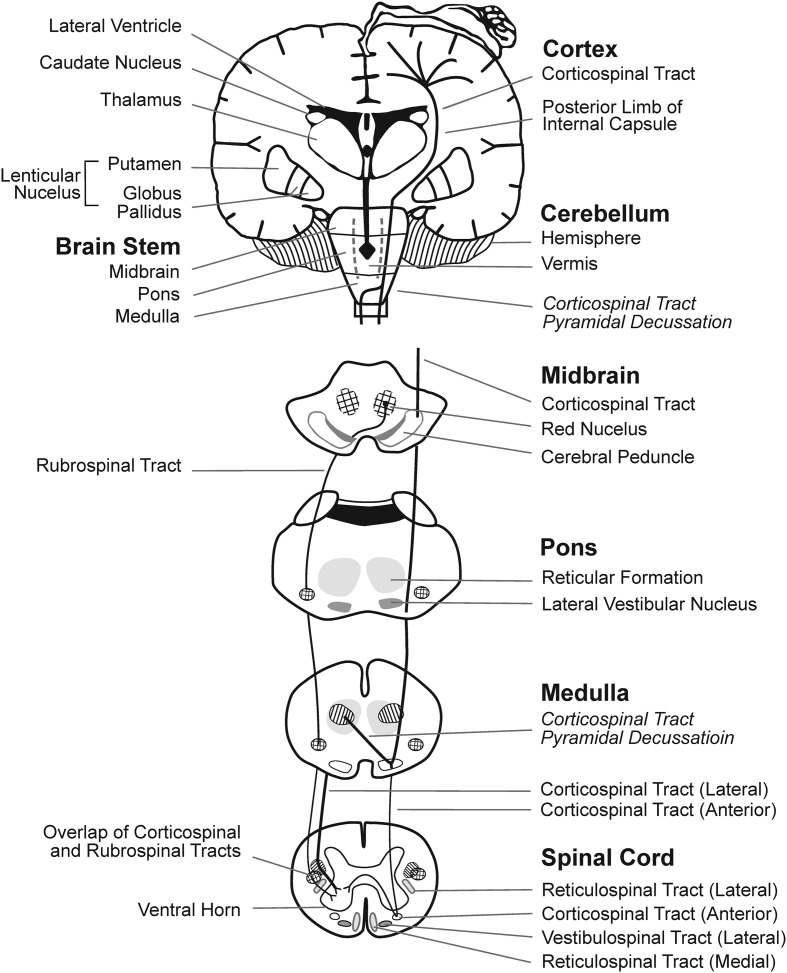
**Brain regions and WM motor tracts affected in spastic, dyskinetic, and ataxic CP**. A portion of the motor homunculus is superimposed on the cortex. Representations of the lateral ventricles, subcortical nuclei, cerebellum, brain stem, and rubrospinal and CSTs are outlined. The vermis of the cerebellum (posterior to the brainstem) is represented by dotted lines. Regions where the reticulospinal and vestibulospinal tracts descend through each layer of the brainstem and the spinal cord are shaded.

There are three main types of CP: spastic, dyskinetic, and ataxic. Spastic CP is the most common, affecting approximately 87% of children with CP, while dyskinetic CP affects approximately 7.5%, and ataxic CP affects approximately 4% of children with CP (Sellier et al., 2016). There is evidence that different types of CP each have primary regions of brain damage linked to characteristic motor deficits, though different types of CP can co-exist. Emerging research indicates that spastic CP is associated with brain damage to cortical motor areas and underlying WM, dystonic CP is associated with damage to basal ganglia, and ataxic CP is associated with damage to cerebellar structures (**Figure 1**). In general, periventricular WM lesions are generally associated with mild and moderate motor impairments of spastic CP with fewer accompanying impairments, whereas brain maldevelopment and cortical/subcortical and basal ganglia lesions are associated with more severe and a greater number of accompanying impairments, such as cognitive and language deficits (Himmelmann and Uvebrant, 2011).

Spastic CP is often linked to damage to the periventricular WM due to hypoxia-ischemia, which may be caused by various maternal or prenatal factors. Spastic CP is further delineated by the distribution of affected limbs: hemiplegia, diplegia, and tetraplegia (also referred to as quadriplegia). Individuals with spastic CP present with varying degrees of weakness, short muscle-tendon unit, spasticity, and impaired SMC. In spastic CP, the severity of motor deficits, the distribution of affected limbs, and the extent to which the motor deficits involve distal to more proximal joints within a limb, are thought to be determined by the severity and extent of the brain injury (Serdaroglu et al., 2004), consistent with anatomical representation of the cortical homunculus and descending fibers (**Figure 1**). More medial brain injury, i.e., closer to the ventricles, results in more mild involvement of distal joints. More extensive injury that extends to lateral portion of periventricular WM results in more severe involvement of both distal and proximal joints. Accordingly, Staudt et al. (2000) found a strong correlation between the extent of the lateral lesion in the posterior semi-coronal plane on MRI and the lower limb motor impairment score (*r* = 0.805, *p* = 0.001). In addition, upper limb involvement is generally associated with a greater degree of lateral damage compared to lower limb involvement. Staudt et al. (2003) found that upper limb dysfunction was observed only in patients with a lateral lesion extent of 23 mm or more, whereas lower limb dysfunction was present with a lateral lesion extent of 20 mm.

Dyskinetic CP is the second most common type of CP, affecting approximately 7.5% of children with CP (Sellier et al., 2016). It is often linked to damage of the subcortical GM, i.e., basal ganglia and thalamus, due to hypoxia-ischemia, as well as hyperbilirubinemia and birth asphyxia (Himmelmann and Uvebrant, 2011). The definition of dyskinetic CP has evolved recently to include dystonic and choreoathetoid CP (Aravamuthan and Waugh, 2016). As noted by Sanger (2006), symptoms of dyskinetic CP include both hyperkinetic and dystonic limb movements that impair function. Currently, it has not been determined whether these movements are random and variable or involve a small number of specific abnormal motor patterns (Sanger, 2006). The severity of motor impairment in dyskinetic CP has been found to be associated with lower volumes of the basal ganglia and thalamus (Laporta-Hoyos et al., 2014). In a study of 18 children with basal ganglia lesions and CP, 13 children had a diagnosis of dyskinetic CP, 12 had a diagnosis of dyskinetic CP with severe fine motor impairment and a GMFCS (Palisano et al., 2008) level of IV or V, representing severe CP with reduced or absent independent mobility (Himmelmann and Uvebrant, 2011).

Ataxic CP is the least common type of CP and is associated with cerebellar vermis injury, cerebellar malformations, and or genetic mutations; ataxic CP is characterized by impaired limb coordination during voluntary movements, as well as balance, stability, and speech impairments (Hughes and Newton, 1992; Schnekenberg et al., 2015; Graham et al., 2016). Few studies have examined the neurologic correlates of ataxic CP. Miller and Cala (1989) examined CT scans of 29 patients with ataxic CP born at term: 7 had simple ataxic CP and 10 had ataxic diplegia, which involves simple ataxia in addition to pyramidal signs of spastic diplegic CP. They identified brain abnormalities in only 18/29 children. Of the 7 children with simple ataxia, 5/7 had cerebral abnormalities and 4/7 had changes in the posterior fossa involving the vermis (Miller and Cala, 1989). Similarly, Bax et al. (2006) found that children with ataxic CP had the highest rate of normal MRI (8 out of 17), suggesting a need for further research utilizing higher resolution neuroimaging in ataxic CP.

Although early brain injury is the primary cause of CP, a recent study using exome sequencing reported that 14% of CP cases had causative single-gene mutations and up to 31% had clinically relevant copy number variations, suggesting a greater role of genetics in the development of brain injury and CP than previously recognized (MacLennan et al., 2015). Genes encoding processes related to catabolism of lipoprotein constituents, procoagulant factors, factors influencing central nervous system injury response, neuronal function (genes for potassium channels), cytoskeleton-interacting proteins, and adaptor proteins involved in intracellular trafficking are targets of further research as potential culprits of genetic causes for CP (Fahey et al., 2016). For the purposes of this review, we will focus primarily on the neurologic correlates of gait abnormalities in CP and the implications for treatment.

Currently, brain imaging using MRI is the gold standard for identifying neural injury in CP. Recently, Fiori et al. (2015) developed an MRI score that correlated with severity of CP. In a review of six studies, including 1065 MRI and CT images in children diagnosed with CP, the most common injuries were WM injury (19–45% of images), GM injury (14–22%), focal vascular insults (10%), malformations (11%), and miscellaneous abnormalities (4–23%) (Reid et al., 2014). WM injury was most common among all CP subtypes, though children with spastic diplegia had the highest rate of WM injury overall.

However, brain injuries in CP are not always visible on MRI; a review by Reid et al. (2014) revealed that abnormal MRI only accounts for about 86% of CP cases, i.e., 14% of children clinically diagnosed with CP show no signs of brain injury on MRI, particularly in children with ataxic CP, as noted above (Bax et al., 2006). These findings may be explained by the limitations of current imaging methodologies, such as insufficient spatial resolution, to reveal micro-lesions (Leonard et al., 2011; Benini et al., 2013). In fact, recent studies show that DTI can better predict motor function deficits from WM damage in the posterior limbs of the internal capsule than conventional MRI (Rose et al., 2007; Benini et al., 2013). Furthermore, a recent publication suggests that the combination of different MRI scans, including volumetric imaging and DTI, can help to identify potential relations between brain lesions and lower limb deficits or gait pathology in children with spastic CP (Meyns et al., 2016b).

Selective vulnerability of developing WM to injury during early phases of vascularization and WM myelination has been widely recognized (Inder and Volpe, 2000; Hoon et al., 2009). Regional differences in the trajectory of early WM development (Dubois et al., 2014; Rose et al., 2014) can influence vulnerability and thus the pattern of brain injury typically seen during the preterm, term, or postnatal periods (Volpe, 2003; Graham et al., 2016). That said, determining the time of onset of brain injury in CP can be difficult to pinpoint. Here, we discuss brain injury among infants based on a preterm (<37 weeks gestation) versus term birth and specify the time of injury when reported (**Table 1**).

**Table 1:** Mechanisms and patterns of brain injuries commonly identified in pre-term and term infants that contribute to CP.

Preterm infants	Term infants
**Mechanisms**	**Mechanisms**
Intraventricular hemorrhage	Hypoxia-ischemia
Hypoxia-ischemia	Inflammation
Inflammation	Infection
Infection	
Postnatal sepsis	Postnatal sepsis
Postnatal brain injury	Postnatal brain injury
Postnatal bilirubin toxicity	
**Patterns of injury**	**Patterns of injury**
Periventricular white mater lesions Cystic periventricular leukomalacia	Border zone (watershed) white matter injury
Non-cystic periventricular leukomalacia Injury to thalamocortical sensory fibers	Combination deep gray matter and white matter injury
Cortical and deep gray matter lesions Reduction in brain volumes	Cystic encephalomalacia Focal infarcts
Cerebellar injury	Cerebellar injury
Brain malformations	Brain malformations

### Brain Injury in Preterm Infants

#### Incidence and Risk Factors in Preterm Infants

Preterm birth is associated with approximately one-third of all CP cases (Oskoui et al., 2013) and approximately 30% of dyskinetic CP (Himmelmann et al., 2009). Brain injury among preterm infants can occur in the prenatal, perinatal, or postnatal period. The timing of the injury and the developmental stage of the brain influences the type of deficit. For example, damage to the brain during the late second trimester, in which the vulnerable process of regional WM myelination of motor tracts occurs, results in lasting motor dysfunction. Preterm birth in instances of PVL was found by Serdaroglu et al. (2004) to correlate significantly with spasticity.

Preterm infants have an increased risk of prenatal hypoxia-ischemia, prenatal and postnatal intraventricular hemorrhage, and PVL, and thus, an increased risk for CP. Complications experienced by preterm infants such as sepsis and necrotizing enterocolitis also increase the risk of CP (Mallard et al., 2013). Indeed, VLBW preterm infants (≤1500 g at birth, ≤32 weeks) who suffer relatively low levels of neonatal inflammation (based on serum levels of C-reactive protein) during the first 2 weeks of life, have a higher risk of neurodevelopmental impairment at 18–22 months (Rose et al., 2016), and neonatal infection has been shown to increase VLBW infants’ risk for WM abnormalities that lead to neurodevelopmental impairment (Rose et al., 2014; Rand et al., 2016). Prenatal brain lesions or cerebral infarction can arise from thrombosis due to inflammation and low blood flow in the placenta, brain, or other organs (Nelson and Blair, 2015).

Pregnancy complications are common in preterm birth (42% of cases, *n* = 92,320) and often contribute to a diagnosis of CP. Such complications include chorioamnionitis (11.2% absolute risk of CP), cervical conization (9.3%), placental abruption (8.7%), placenta previa (8.3%), congenital malformation (8.3%), prolonged rupture of membranes (7.5%), intrauterine growth restriction (7.1%), unspecified bleeding (6.8%), multiple births (5.7%), and pre-eclampsia (3.7%) (Trønnes et al., 2014). Further, maternal infections during pregnancy (excluding the common cold, coughs, etc.) have been reported in 29.6% (118 of 400) of cases of CP (Bax et al., 2006). Specifically, 19.2% of the mothers reported a urinary tract infection during pregnancy and 15.5% of women reported taking antibiotics during pregnancy. Twins have a greater risk of CP compared with singletons, especially if the pair have growth discordance (Scher et al., 2002). In addition, the *in utero* death of one twin leaves the surviving infant at a tenfold increased risk for CP, as dispersed intravascular coagulation and emboli in the vascular anastomoses of the twin placenta likely contribute to prenatal cerebral injury in the surviving twin (Scher et al., 2002; Nelson and Blair, 2015). Marked fetal growth restriction with presence of major birth defects is also associated with an increased risk of CP (Decouflé et al., 2001; Blair and Nelson, 2015). In addition, sex differences in neurodevelopment have been identified: the incidence of moderate-to-severe CP was found by Hintz et al. (2015) to be 50% higher in males (10.7%) than in females (7.3%) in extremely low-birthweight (<1000 g) preterm infants. They found no measurable risk factors or events, including a diagnosis of intraventricular hemorrhage on ultrasound, that explained the sex differences in neurodevelopmental outcomes. Findings indicate a distinct disadvantage in male infants for developing CP that is exacerbated in the preterm population.

#### Mechanisms of Injury in Preterm Infants

The regions of the brain adjacent to the lateral ventricles are especially susceptible to hypoxia-ischemia, i.e., insufficient blood flow combined with reduced concentration of oxygen in arterial blood, during prenatal development. In the preterm brain, the intrinsic architecture of the arterial border culminates in end zones that lie within the WM. This physiologic propensity to develop ischemia is further perturbed by impaired regulation of cerebral blood flow and metabolic needs during development (Khwaja and Volpe, 2008). Oligodendrocytes in the periventricular WM actively proliferate and myelinate during the third trimester (27–40 weeks gestation), and their high metabolic demand renders them vulnerable in preterm infants (Raybaud, 1983; Talos et al., 2006).

The increased risk for infection/inflammation (including ischemia-induced inflammation) during fetal development also contributes to the mechanism of injury in PVL. Upregulation of pro-inflammatory cytokines and activation of microglia within immature WM results in damage to the vulnerable premyelinating oligodendrocytes. This cell-specific damage results in WM hypo-myelination, and necrotic microscopic lesions lead to proliferation of glial cells in response to injury, a common finding in non-cystic PVL and among preterm children with CP (Graham et al., 2016). Mallard et al. (2013) suggested that the inflammation-induced opening of connexin hemichannels plays a pivotal role in initiating a cycle of excessive ATP release, over-activation of purinergic receptors on microglia and astrocytes, and subsequent brain damage.

After initial injury by hypoxia-ischemia and infection/inflammation, excitotoxicity and free radical attack by reactive oxygen and nitrogen species are the main downstream mechanisms of injury in PVL. Current evidence supports that oligodendroglial development is susceptible to oxidative attack within a maturation-dependent window of vulnerability. This window of vulnerability is due mainly to delayed development of antioxidant enzymes and the acquisition of iron for oligodendrocyte differentiation. Human brain studies have shown a delay in the development of superoxide dismutase (antioxidant) enzymes, e.g., manganese superoxide dismutase, copper/zinc superoxide dismutase, and catalase (Folkerth et al., 2004). Further, observations show that developing WM uses iron both for oligodendrocyte differentiation (Connor and Menzies, 1996) and to convert hydrogen peroxide to its hydroxyl radical, thereby resulting in the increased free iron levels in the cerebrospinal fluid of children with posthemorrhagic ventricular dilation (Savman et al., 2001). Excitotoxicity likely leads to injury to premyelinating oligodendrocytes by promoting Ca^2+^ influx and generation of reactive oxygen and nitrogen species. Glutamate is capable of inducing maturation-dependent death of premyelinating oligodendrocytes by receptor-mediated mechanisms *in vivo* (Khwaja and Volpe, 2008). Premyelinating oligodendrocytes contain glutamate receptors that, when excessively activated, lead to cell injury. The excess glutamate comes from oligodendrocytes that express AMPA/kainate-type glutamate receptors and NMDA receptors, the over-activation of which results in cell death (Káradóttir and Attwell, 2007; Khwaja and Volpe, 2008). In addition, the AMPA/ kainate receptors have been found to be upregulated in premyelinating oligodendrocytes rather than in mature oligodendrocytes (Rosenberg et al., 2003; Jensen, 2005).

#### Patterns of Injury in Preterm Infants

**FIGURE 2:**
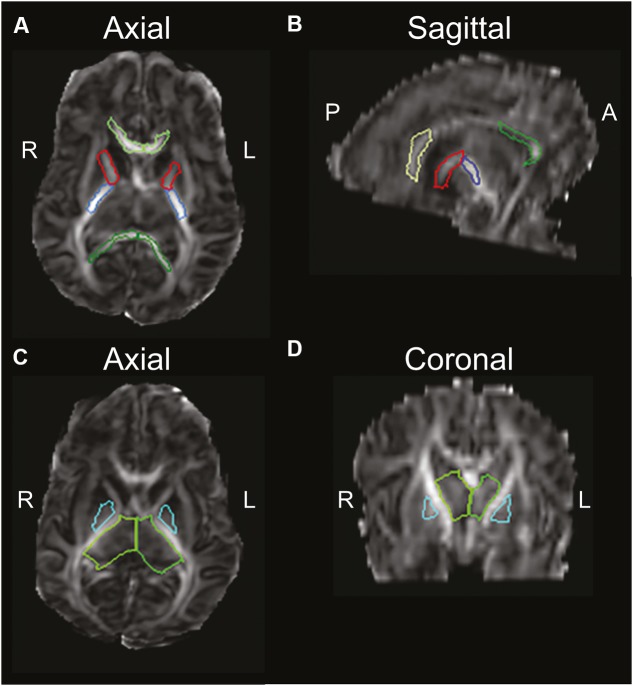
**Diffusion-weighted image of an infant, highlighting brain regions of interest commonly affected in CP, using the semi automated JHU neonatal atlas (http://cmrm.med.jhmi.edu/cmrm/Data_neonate_atlas/atlas_neonate.htm)**. Axial view **(A)** and sagittal view **(B)** of the genu (light-green) and splenium (dark-green) of the corpus callosum, and anterior (red) and posterior (blue) limbs of the internal capsule. An axial view **(C)** and coronal view **(D)** highlight the thalamus (green) and globus pallidus (cyan). A, anterior; P, posterior; R, right; L, left. Figure reprinted with permission from Rose et al. (2015) Pediatric Research. http://www.nature.com/pr/journal/v78/n6/fig_tab/pr2015157f2.html

Patterns of brain injury on MRI among preterm children with CP who suffered hypoxia-ischemia revealed 51/104 (49%) had signs of PVL or signal abnormalities in the periventricular WM (Sie et al., 2000). Indeed, periventricular WM lesions are the most common injury among preterm children, followed by cortical and deep GM lesions (Hoon et al., 2009). **Figure 2** highlights the brain regions and WM tracts commonly affected in CP.

The WM tracts essential for motor function descend near the periventricular region through the posterior limbs of the internal capsule; therefore, periventricular WM injury may result in impaired motor function and a diagnosis of CP (**Figure 2**). The CST is the major WM tract responsible for voluntary movement. About 40% of its fibers originate from the primary motor cortex in the precentral gyrus; the other 60% originate in the supplementary motor area, the premotor cortex, the somatic sensory cortex, the parietal lobe, and the cingulate gyrus. These fibers descend through the corona radiata and posterior limb of the internal capsule to reach the brainstem, where 80% of the fibers decussate at the spinomedullary junction and continue their descent contralaterally as the lateral CST (Mtui et al., 2016, p. 169–178). Damage to developing WM in the CST is considered a major mechanism for motor dysfunction in preterm children with spastic CP (Roelants-van Rijn et al., 2001; Rose et al., 2007).

A growing body of evidence from DTI and tractography studies suggests that thalamocortical sensory fibers are also particularly vulnerable during the preterm period. The thalamocortical sensory fibers extend from the thalamus to the primary and secondary somatosensory areas, terminating in cortical layers of the lateral postcentral gyrus. Injury to these WM fibers can impair motor and somatosensory function in children with CP (Hoon et al., 2002, 2009; Rose et al., 2011; Tsao et al., 2015). Hoon et al. (2002, 2009) found that the severity of injury in the thalamocortical sensory pathways correlated to the severity of deficits in sensorimotor function in children with CP born preterm. Similarly, abnormalities assessed using DTI in preterm infants were seen in both CST and thalamocortical projections to the somatosensory cortex, and both tracts were associated with underlying pathology on conventional MRI (Lennartsson et al., 2015).

Cortical GM and BGTL may occur in preterm children as a result of hypoxia-ischemia. Injury to the GM has been linked most frequently to spastic quadriplegic CP and dyskinetic CP (Reid et al., 2014).

Preterm children may also have long-term reductions in regional brain volumes of the sensorimotor cortex, which are associated with poorer cognitive and visuomotor outcomes (Peterson et al., 2000).

#### Postnatal Brain Injury in Preterm Infants

Preterm children may be more susceptible to brain injury or insult during the postnatal period. Postnatal sepsis has been associated with non-cystic PVL in preterm children who later develop CP (Volpe, 2008). Male infants under 1 year of age, who were born weighing less than 1500 g, are at the greatest risk for developing CP following postnatal sepsis (Blair and Stanley, 1982). Other causes of postnatal brain injury resulting in CP include cerebrovascular accidents, head trauma, and epilepsy (Arens and Molteno, 1989; Australian Cerebral Palsy Register Report [ACP], 2013). Postnatal intraventricular hemorrhage among preterm children with CP can also result in cerebellar injury, which may contribute to diminished walking and verbal abilities, a higher incidence of epilepsy, and visual impairment (Kitai et al., 2015). Postnatal bilirubin toxicity is a complication among preterm infants that may be related to GM injury. The exposure to even moderate levels of unconjugated bilirubin may cause damage to the developing central nervous system, specifically the basal ganglia and cerebellum. Brain lesions identified on MRI following extreme hyperbilirubinemia have been linked to dyskinetic CP, though this is rare in developed countries (Rose et al., 2014) as a result of effective monitoring and treatment for hyperbilirubinemia in preterm infants.

### Brain Injury in Term-Born Infants

#### Incidence and Risk Factors in Term-Born Infants

The majority of CP cases (60%) are acquired in the term-born population during the perinatal period (?). Approximately 70% of dyskinetic CP cases are linked to insults acquired in the term-born infant (Himmelmann et al., 2009). The risk factors for CP among term-born infants in developed countries include: placental abnormalities, birth defects, low birthweight for gestational age, meconium aspiration, instrumental/emergency Cesarean delivery, neonatal seizures, respiratory distress syndrome, hypoglycemia, and neonatal infection (Review: McIntyre et al., 2013). Neonatal infections include chorioamnionitis (which has been associated with WM damage), neurotropic virus infection, cytomegalovirus infection, and maternal urinary tract infection (Ahlin et al., 2013). Infection-related factors have been shown to be independent risk factors for spastic hemiplegic CP, but not for spastic diplegia, tetraplegia, or dyskinetic CP in term-born infants (Ahlin et al., 2013). A recent review of 23 studies on perinatal risk factors of CP refutes birth asphyxia as a primary cause of CP (Ellenberg and Nelson, 2013).

Although perinatal ischemic stroke accounts for less than 5% of all term-born CP cases (Wu et al., 2011), it accounts for 30% of hemiplegic CP cases (Wu et al., 2006). Embolization from the placenta near the time of delivery, a period characterized by hypercoagulability, has been suggested as a cause of perinatal stroke. Larger vessels are more frequently involved, especially the left middle cerebral artery (Nelson and Blair, 2015) resulting in left-sided brain lesions, which correspond to right-sided motor deficits.

#### Mechanisms of Injury in Term-Born Infants

Neonatal encephalopathy due to presumed hypoxia-ischemia remains an important clinical problem in term-born infants, with placental pathology providing insight to the underlying mechanisms. Among term-born infants, an elevated nucleated red blood cell count in the placenta was significantly related to watershed (border zone) WM brain injury (Frank et al., 2016). A watershed (border zone) injury arises from prolonged, partial ischemia of the WM between two major arteries, commonly the anterior–middle and posterior–middle cerebral arteries. Further, infants with small infarcts or an elevated nucleated red blood cell count on placental pathology were less likely to develop BGTL injury following perinatal hypoxia-ischemia, and were more likely to have a favorable neurodevelopmental outcome. Frank et al. (2016) have posited that both small infarcts and elevated nucleated red blood cell counts, which reflect adverse intrauterine conditions, might be involved in a pathway to preconditioning resistance against acute brain injury, i.e., neuroprotective factors. The mechanisms linking infection and cerebral ischemia are still largely undetermined, but inflammation is thought to exacerbate the natural prothrombotic state present during normal pregnancy, and stimulates coagulation (Grau et al., 1995; Ahlin et al., 2013).

#### Patterns of Injury in Term-Born Infants

In a study of 173 term-born infants with neonatal encephalopathy, the pattern of brain injury on MRI revealed 45% of newborns had predominantly a watershed (border zone) pattern of WM injury, 25% had BGTL injury and 30% had normal MRI (Miller et al., 2005). The predominant region of injury was often accompanied by lesser damage to other regions. For example, 31% of newborns with the watershed (border zone) predominant pattern had some BGTL injury, and 45% of newborns with the BGTL predominant pattern had total brain injury. The BGTL predominant pattern was significantly associated with severe neonatal signs, encephalopathy, seizures, and severe motor and cognitive outcome at 30 months. Among term-born children with CP, Sie et al. (2000) found that nearly 20% had bilateral BGTL injury involving the putamen and thalamus. Among these children, one-third had additional globus pallidus lesions and about half had additional hippocampal lesions and WM abnormalities. These WM abnormalities were unique from that of PVL and varied from ventricular dilatation to diffuse and patchy mild WM signal changes. Brain malformations, such as congenital microcephaly, and GM lesions are more often seen in term-born children than preterm children with CP (Miller et al., 2005; Krägeloh-Mann and Horber, 2007).

Another pattern of injury present among term-born children with CP and hypoxic-ischemic injury is multicystic encephalopathy, which involves multiple large cystic cavities in the WM separated from each other by membranes and is associated with periventricular, subcortical, and cortical damage (Sie et al., 2000).

Focal vascular insults are seen predominantly in term-born children with hemiplegic CP, whereas malformations tend to be associated with ataxic, quadriplegic, and diplegic CP (Reid et al., 2014). Of these malformations, schizencephaly occurs more often in patients with spastic hemiplegia than in any other CP subtype (Kulak et al., 2011).

#### Postnatal Brain Injury in Term-Born Infants

Among 3135 individuals with CP born from 1993–2006, 5.6% of individuals acquired a brain injury due to a recognized event more than 28 days after birth, with the predominant cause listed as cerebrovascular accident (34.2%) (Australian Cerebral Palsy Register Report [ACP], 2013). The cerebrovascular accident was either spontaneous, associated with surgery, or due to complications of cardiac defects. Other causes of postnatal brain injury in term-born infants leading to CP include head trauma, cerebral infections, prolonged seizures, and respiratory arrest or anoxia (Blair and Stanley, 1982; Arens and Molteno, 1989). Approximately 7% of dyskinetic CP cases are linked to brain injuries incurred during the postnatal period (Kyllerman, 1982).

## Neuromuscular Deficits of Cerebral Palsy

Neuromuscular deficits differ among spastic, dyskinetic, and ataxic CP and involve abnormal motor drive, muscle tone, motor patterns, and coordination caused by the original brain injury. In addition, subsequent sensorimotor and musculoskeletal changes result from chronic abnormal muscle activation, biomechanical imbalance around joints, neglect, and/or disuse. These factors, combined with rapid limb growth and increasing body weight in children, contribute to gait abnormalities in CP (Meyns et al., 2016a).

### Neuromuscular Deficits of Spastic CP

In spastic CP, neurological injury to the CST results in four interrelated neuromuscular deficits: muscle weakness, shortened muscle-tendon unit, spastic and passive resistance to stretch, and impaired SMC. These deficits arise from brain injury and subsequent changes in the motor unit, muscle growth, and muscle fiber composition. Further involvement of sensory-motor regions can impair proprioception and motor function (Hoon et al., 2009). Together, these neuromuscular deficits result in the gait abnormalities commonly seen in children with spastic CP.

The motor unit is the functional unit of the motor system, consisting of a single motor neuron, the neuromuscular junction, and the muscle fibers innervated by the motor neuron. In the gastrocnemius, for example, a motor unit consists of a single motor neuron with approximately 2000 associated muscle fibers, whereas smaller muscles in the hand that are responsible for fine motor control have a much smaller muscle fiber-to-motor neuron ratio. Rose and McGill (2005) found reduced neuromuscular activation and motor-unit firing rates in the medial gastrocnemius and tibialis anterior in spastic CP. The altered neural input to muscle has been associated with altered muscle growth, fiber type and size variability, sarcomere length, as well as altered collagen, fat, and extracellular matrix composition. These changes in skeletal muscle morphology contribute to functional changes in muscle strength, the length of the muscle-tendon unit, and the reflexive and passive resistance to stretch.

Muscle fiber development and growth rely on a number of neuronal, nutritional, and hormonal factors, as well as initial and repeating patterns of muscle use (Kimball and Jefferson, 2010; Braun and Gautel, 2011; Gundersen, 2011). Muscle growth increases rapidly during the prenatal and neonatal periods. For example, the sartorius muscle fibers double in diameter between mid-gestation and term (Moore et al., 1971), and accelerated growth in overall muscle fiber diameter has been noted between 35 weeks of gestation and term (Schloon et al., 1979). Thus, preterm delivery may interfere with early muscle growth, and compromised nutritional status in the preterm infant likely contributes to poor skeletal muscle growth (Thorn et al., 2009). It has been shown that muscle size is reduced in spastic CP (Barber et al., 2011), and muscle volume is especially reduced in the gastrocnemius and semitendinosus, both of which are critical for gait (Smith et al., 2011). Recent work by Dayanidhi and Lieber (2014) suggests that satellite cells also play an important role in muscle growth. Muscle biopsies from children with CP had 60–70% fewer satellite cells when compared to that of age-matched, TD children, which may contribute to muscle contracture in CP.

Muscle biopsy reveals an increased proportion of type-1 muscle fibers and an increased variability in muscle fiber diameter in the muscles affected by spastic CP, likely as a result of prolonged low-frequency motor unit firing rates (Rose et al., 1994). Complex changes occur at both the level of the fiber and the gross muscle, including an altered transcriptional profile, increased sarcomere length, stiffer extracellular matrix, and reduced overall muscle length, all of which contribute to muscle contracture (Smith et al., 2011). Other changes in spastic muscle in CP include increased collagen (Booth et al., 2001) and fat content (Johnson et al., 2009). Although spastic muscle contains a larger amount of extracellular matrix material compared with normal muscle, the quality of the extracellular matrix is much lower, contributing to the overall increased stiffness (Lieber et al., 2003).

#### Weakness in Spastic CP

Children with spastic CP suffer from significant weakness that contributes to abnormal posture and movement (Brown et al., 1991; Damiano et al., 1995; Rose and McGill, 2005; Barber et al., 2012; Noble et al., 2014). Studies suggest that the loss of excitatory motor signals descending in the CST results in reduced muscle activation and reduced muscle size, which is aggravated further by pathological changes in the elasticity of the muscle (Brown et al., 1991; Wiley and Damiano, 1998; Engsberg et al., 2000). MVC of the quadriceps, plantar flexors, and dorsiflexors are significantly reduced in CP (Damiano et al., 2002; Elder et al., 2003; Rose and McGill, 2005; Stackhouse et al., 2005; Barber et al., 2012). Barber et al. (2012) found a 33% lower ankle plantarflexion torque in children with spastic CP compared to their TD peers. This reduced ankle plantarflexion torque was partially explained by 37% smaller medial gastrocnemius muscle and 4% greater antagonistic co-contraction.

It has been posited that individuals with spastic CP do not develop sufficient tension frequently enough to encourage normal muscular growth (Noble et al., 2014). Noble et al. (2014) found that the medial and lateral gastrocnemius, soleus, tibialis anterior, rectus femoris, semimembranosus, and semitendinosus of patients with CP had reduced volumes compared to TD children, even when adjusted for body mass. This lack of growth may be mediated through reduced muscle and neurotrophic factors that are released in response to neuronal activation, acetylcholine release, and contraction (Gough and Shortland, 2012), further contributing to weakness. Affected muscles in spastic CP have substantially reduced neuromuscular activation and strength (Damiano et al., 2001; Rose and McGill, 2005; Stackhouse et al., 2005) and an inability to sufficiently recruit and drive motor-units at higher firing rates (Rose and McGill, 2005). Further, musculoskeletal manifestations progress as skeletal growth out-paces muscle growth, leading to reduced muscle volumes associated with weakness (Noble et al., 2014). Muscle endurance is also reduced in CP compared to TD individuals; specifically, adolescents with CP have a reduced capacity to endure activities at similar relative loads compared with TD adolescents (Eken et al., 2014).

Common surgical procedures for CP negatively affect muscle strength. For example, selective dorsal rhizotomy reduces antigravity support that may have been provided by spasticity (Giuliani, 1991), surgical muscle-lengthening or tendon transfer decrease muscle force production, intrathecal baclofen directly weakens the muscle to reduce spasticity (Hallett, 2000), and orthoses or serial casting may exacerbate weakness due to immobilization. Similarly, BoNT-A weakens the injected muscles in order to reduce spasticity (Hallett, 2000). However, a more recent study on the effects of BoNT-A suggests no decrease in long-term muscle strength at 6 weeks or 6 months after a one-time injection of BoNT-A in children with CP (Eek and Himmelmann, 2016).

While the neural correlates of muscle weakness are not well studied, Meyns et al. (2016b) recently found a correlation between asymmetry in strength and asymmetry in the CST ADC calculated from DTI (*r* = 0.639, *p* < 0.034) of the segment of the CST that runs through the posterior limb of the internal capsule (CST_PLIC_). Thus, greater asymmetry in strength was associated with a greater ADC asymmetry of the CST_PLIC_. Further research is needed to clarify the brain structure-function relations underlying weakness in spastic CP to develop effective treatments.

#### Short Muscle-Tendon Unit in Spastic CP

Impaired muscle growth and muscle fiber changes result in a shortened muscle-tendon unit in the muscles affected by spastic CP. The failure of muscle growth to keep pace with bone growth is most evident in the bi-articular muscles, e.g., the gastrocnemius, hamstrings, and rectus femoris, and contributes to joint contractures and gait abnormalities such as toe-walking and flexed-knee gait.

Among TD children ages 5–12 years, the medial gastrocnemius has been shown to demonstrate 20% longitudinal growth compared to 80% cross-sectional growth of muscle fibers (Benard et al., 2011). In the CP population, while muscles on the affected side of children with hemiplegic CP were smaller compared to the unaffected side, the altered morphology was not due to a decrease in fascicle length, i.e., longitudinal growth, but rather a lack of cross-sectional growth (Barrett and Lichtwark, 2010). Similarly, Shortland et al. (2001) concluded that a smaller medial gastrocnemius in ambulatory children with spastic diplegia was not due to reduced muscle fiber length, but rather to shortened aponeuroses of the pennate muscle via reduced muscle fiber cross-sectional diameter. Shortened muscle tendon units in spastic CP result in part from reduced muscle growth. Barber et al. (2016) found that normalized medial gastrocnemius muscle growth rate was significantly less in children with unilateral CP compared to children with bilateral CP and TD children. Treatment with muscle growth factors has not been studied to date, however, it has potential to increase muscle size and length and warrants investigation as a treatment for CP. Smith et al. (2009) assessed the transcriptional profile in biopsies of spastic muscle in six children with CP and compared it with that of two typically developing children. They noted competing upregulation of both insulin-like growth factor 1 and myostatin, as well as an aberrant regulation of excitation-contraction coupling genes. More research is needed to better characterize the adaptations occurring at a molecular level, but these studies point to growth factors as an avenue of research for novel therapies.

The short muscle-tendon unit also likely contributes to weakness. The active force generated by muscle is a function of the number of cross-bridges formed, which depends on the extent of myofilamentary overlap. Muscle force is maximal at intermediate muscle lengths with optimal myofilament overlap and declines at shorter and longer relative lengths (Gordon et al., 1966). Thus, short muscles with lengthened sarcomeres, as has been found in spastic muscle in CP (Lieber et al., 2004), may result in an inefficient overlap of myofilaments, thereby contributing to weakness. Research on the neural correlates of short muscle-tendon unit is needed, considering the impact of reduced descending excitatory signals on muscle growth, muscle-to-bone growth rate discrepancy, and muscle fiber diameter.

#### Spasticity in Spastic CP

Resistance to muscle stretch in children with spastic CP is primarily due to two factors: neural-mediated reflex stiffness (muscle spasticity) and passive muscle stiffness. Spasticity and passive resistance to muscle stretch particularly influence bi-articular muscles, such as the rectus femoris, hamstrings, and gastrocnemius, which require greater excursion across two joints.

Spasticity has been defined as “a velocity-dependent increase in muscle tone with exaggerated tendon jerks, resulting from hyper-excitability of the stretch reflex” (Mayer, 1997). Loss of descending inhibition of the stretch reflex pathway to affected muscles in spastic CP may result in increased sensitivity to stretch (Rose and McGill, 1998).

In addition to the velocity-dependent sensitivity to stretch (Sanger et al., 2003), spasticity may also be position-dependent. Wu et al. (2010) found that evaluating patients at different velocities and positions helped to distinguish passive stiffness from spasticity. The delayed catch angle at higher velocities may be due to position change, as the joint is moved into a stiffer position.

Although muscle spasticity is a primary symptom of spastic CP, objective quantification has been challenging (Bar-On et al., 2014b). Recent data on the profile of imposed muscle accelerations, including the muscle length–velocity relationship, hold promise for quantifying spasticity. Bar-On et al. (2014c) quantified integrated biomechanical (joint position and torque) and electrophysiological (surface EMG) signals of manually performed passive stretches on the medial hamstrings and gastrocnemius and found that measurement reliability was moderately high for both muscles, and spasticity parameters were significantly higher in children with spastic CP than in TD children. Similarly, biomechanical parameters quantifying the neural and non-neural contributions to ankle joint torque were measured during manually applied passive stretches to the gastroc-soleus in children with spastic CP, and the parameters based on modeling of passive muscle stiffness and viscosity were able to detect a significant decrease in spasticity (*p* = 0.012) following BoNT-A in 53 children with spastic CP (Bar-On et al., 2014a). This type of musculoskeletal modeling combined with spasticity measurement may allow for individually tailored spasticity treatments. Further, investigation of fast and slow passive rotations imposed during manual and motorized assessments may yield greater insights into the development of movement profiles to better mimic spasticity imposed during functional tasks such as walking (Sloot et al., 2016).

Increased passive muscle stiffness has been demonstrated in spastic CP. Lee et al. (2016) used shear wave elastography to measure muscle stiffness in eight children with hemiplegic CP and found that the more-affected limb had greater muscle stiffness than the less-affected limb. The increased passive mechanical stiffness accounted for nearly all of the measurable increase in joint stiffness (Lieber et al., 2004), suggesting that spastic muscles have an altered resting sarcomere length and altered cellular elastic modulus. Smith et al. (2011) also found that the increased stiffness of hamstring contractures in children with spastic CP compared to age-matched, TD children was likely due to increased stiffness of the extracellular matrix and increased *in vivo* sarcomere length.

Neural correlates of spasticity are emerging. Serdaroglu et al. (2004) found PVL to be associated with spasticity. Specifically, spasticity was found in 54 of 69 participants with body PVL and in 7 of 20 without body PVL. Body PVL was defined as PVL injury in the WM adjacent to the middle region of the lateral ventricle. In addition, lower volume of the total corpus callosum as well as lower volume in the posterior aspect of the central corpus callosum correlated to increased incidence of spasticity (Serdaroglu et al., 2004). Meyns et al. (2016b) recently found a correlation between asymmetry in spasticity and asymmetry in the CST ADC calculated from DTI (*r* = 0.608, *p* < 0.048). Further study will clarify neural correlates and the mechanism underlying spastic and passive resistance in muscle stretch and guide more effective treatment.

#### Impaired Selective Motor Control in Spastic CP

Impaired SMC is defined as an “impaired ability to isolate the activation of muscles in a selected pattern in response to demands of a voluntary posture or movement” (Sanger et al., 2006). Impaired SMC occurs when flexor or extensor synergies interfere with isolated joint movements, resulting in impaired functional movements, such as gait (Rose, 2009; Cahill-Rowley and Rose, 2014). Children with mild to severe spastic CP consistently demonstrate co-activation of the quadriceps and gastrocnemius on EMG, distinguishing spastic CP from idiopathic toe walking (Rose et al., 1999; Policy et al., 2001). Individuals with CP demonstrate reduced complexity of neuromuscular control during gait compared with unimpaired individuals, as determined by the calculation of the muscle synergies during gait (Steele et al., 2015).

Recent studies suggest that spared “extrapyramidal” motor tracts, such as the rubrospinal and reticulospinal tracts, may provide imperfect compensation in recovering motor function. The rubrospinal tract originates in the red nucleus, crosses to the other side of the midbrain, and enters the spinal cord adjacent to the lateral CST. It is thought to mediate flexion and extension movements (Mtui et al., 2016, p. 324–337) and is more developed in infants than TD children and adults. However, rubrospinal tract WM development assessed using DTI was reported to be increased in acute and chronic stroke, yielding characteristic impaired SMC movement patterns post-CST injury (Yeo and Jang, 2010; Rüber et al., 2012). Cortical mapping with DTI in stroke patients suggests that a loss of SMC is also associated with increased overlap of joint representation in the sensorimotor cortices (Yao et al., 2009). The reticulospinal tract originates in the reticular formation in the midbrain and descends to act on the motor neurons supplying the trunk and proximal limb flexors and extensors (Mtui et al., 2016, p. 324–337). It is also thought to modulate pain and influence muscle tone. Current research suggests that infants rely on both the reticulospinal tract and CST for movements, while adults rely primarily on the CST (Forssberg, 1985; Yamaguchi and Goto, 2006). Loss of efferent motor signals descending in the CST provides both excitatory and inhibitory input to subcortical nuclei. Therefore, disruption of these signals may reduce normal inhibition that contributes to SMC. Further studies are needed to characterize development and function of the rubrospinal and reticulospinal tracts, as well as their response to CST injury in spastic CP and their potential for plasticity for compensatory signal transduction and motor function.

Impaired SMC has been assessed using the SCALE, an observation-based measure for children with spastic CP (Fowler et al., 2009). The SCALE has been proven to reliably and systematically quantify the SMC of various joints involved in children with CP. In addition, SCALE scores for children with spastic CP correlate with the GMFCS (Fowler et al., 2009). Further study will clarify neural correlates and the mechanism underlying impaired SMC in spastic CP and guide more effective treatment (Rose, 2009).

### Neuromuscular Deficits in Dyskinetic CP

Dyskinetic CP is characterized by involuntary hyperkinetic or repetitive dystonic limb movements that impair function (Sanger, 2006). More specifically, these movements are dystonic or choreoathetotic in nature: involuntary, uncontrolled, recurring, and occasionally stereotyped, in which the primitive reflex patterns predominate and muscle tone fluctuates (Sankar and Mundkur, 2005; Aravamuthan and Waugh, 2016). Dystonia is a movement disorder in which involuntary sustained or intermittent muscle contractions causes twisting and repetitive movements, abnormal postures, or both (Sanger et al., 2003), and choreoathetosis presents as a mix of chorea and athetosis. Chorea is defined as an ongoing random-appearing sequence of one or more discrete involuntary movements or movement fragments, while athetosis is a slow, continuous, involuntary writhing movement that prevents maintenance of a stable posture (Sanger et al., 2010).

Sanger (2006) found that children with dyskinetic CP had a significantly reduced signal-to-noise ratio compared with TD children, indicating increased movement variability. This finding is consistent with the hypothesis that inadequate removal of noisy signals causes the movement disorder in dyskinetic CP (Sanger, 2006). Most measures of dyskinetic CP have focused solely on dystonia, ignoring choreoathetotic movements. However, Monbaliu et al. (2016) created a scale to measure both dystonia and choreoathetosis in dyskinetic CP, the “Dyskinetic Impairment Scale.” The Dyskinetic Impairment Scale can assess separate movement subscores and has been shown to have high inter-rater reliability (Monbaliu et al., 2016). Further study of neuromuscular deficits in dyskinetic CP will clarify their impact on gait and guide more effective treatment.

### Neuromuscular Deficits in Ataxic CP

Ataxic CP is characterized by hypotonia, impaired limb coordination, balance, and stability (Hughes and Newton, 1992; Schnekenberg et al., 2015; Graham et al., 2016). Impaired balance is well recognized in ataxic CP, however, the scope of other neuromuscular deficits is not well studied. Postural balance deficits assessed on center-of-pressure force plate measures were identified in 8/23 children diagnosed with spastic diplegic CP and gait abnormalities (Rose et al., 2002), suggesting mixed forms of CP. However, the degree to which primary cerebellar balance deficits versus biomechanical factors, such as foot contact area and base of support impair balance in children with CP is not well studied. The vermis of the cerebellum (**Figure 1**) is thought to mediate posture and balance, and it is in this region that the “extrapyramidal” vestibulospinal tract originates. The vestibulospinal tract is comprised of the medial and lateral vestibulospinal tracts. The lateral vestibulospinal tract innervates paravertebral extensors and proximal limb extensors which function to counteract the force of gravity and control posture and balance (Mtui et al., 2016 p. 324–337). Recent studies of treatment to improve balance in children with spastic and ataxic CP are promising and include the application of cerebellar transcranial direct current stimulation, in combination with treadmill training (Lazzari et al., 2016).

## Influence of Neuromuscular Deficits on Gait in Cerebral Palsy: Treatment Implications

Neuromuscular deficits of spastic, dyskinetic, and ataxic CP differ and therefore influence gait in different ways. Neuromuscular deficits include abnormal motor drive, muscle tone, motor patterns, coordination, and sensorimotor impairments caused by the original brain injury. Subsequent sensorimotor and musculoskeletal changes also contribute to gait abnormalities in children with CP.

### The Influence of Neuromuscular Deficits in Spastic CP

**FIGURE 3:**
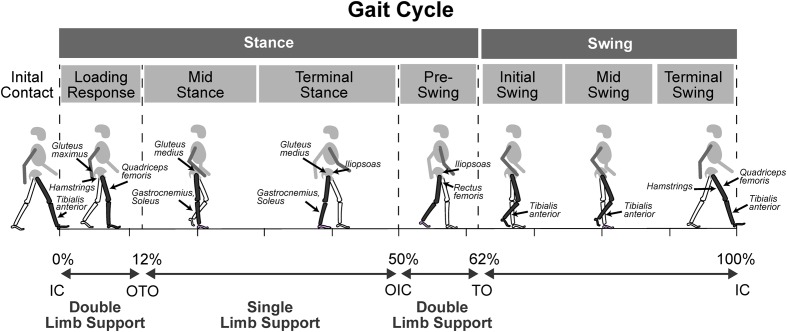
**The gait cycle showing muscles that are influenced by neuromuscular deficits and contribute to gait abnormalities in spastic CP, in relation to phases of the gait cycle**. IC, initial contact; OTO, opposite toe-off; OIC, opposite foot initial contact; TO, toe-off.

The neuromuscular deficits of spastic CP, including muscle weakness, short muscle-tendon length, spasticity, and impaired SMC influence gait in different but predictable ways and contribute to common gait abnormalities such as plantar-flexed gait, flexed-knee gait, or a stiff-knee gait. Plantar-flexed gait includes both ‘drop-foot’ gait where weakness of the tibialis anterior prevents adequate dorsiflexion in swing phase, as well as equinus gait which arises from short and/or spastic plantar-flexors and impacts both stance and swing phases of the gait (**Figure 3** and **Table 2**). In both cases there is typically forefoot initial contact and disruption of the normal heel-toe progression of gait. The plantarflexed position of the ankle causes an early and ‘double bump’ plantarflexion moment that reduces forward momentum. Additionally, the plantarflexed position results in reduced foot contact area in stance that can impair balance.

**Table 2:** Neuromuscular deficits and their contributions to different gait abnormalities in spastic cerebral palsy, in terms of the muscles affected and the timing during the gait cycle.

Neuromuscular deficit	Muscle groups	Gait cycle event	Gait abnormality
**Weakness**	Ankle Dorsiflexors: *tibialis anterior*	IC, Swing	Foot-slap, Drop-foot
	Ankle Plantar flexors: *gastrocnemius, soleus*	Single limb support	Uncontrolled forward tibial rotation → increased hip and knee flexion
			Poor push-off mechanics→ reduced knee flexion in swing
	Knee Extensors: *quadriceps femoris*	IC – Midstance	Increased knee flexion
	Hip Extensors: *gluteus maximus, hamstrings*	IC – Midstance	Increased hip flexion
	Hip Flexors: *iliopsoas*	Preswing	Reduced peak knee flexion in swing
	Hip Abductors: *gluteus medius*	Single limb stance	Contralateral pelvic drop and ipsilateral trunk lean

**Short Muscle-tendon**	Ankle: *gastrocnemius, soleus*	Throughout gait cycle	Increased ankle plantar flexion
	*posterior tibialis*	Throughout gait cycle	Ankle equinovarus
	Knee: *hamstrings*	Stance, Terminal swing	Increased knee flexion
	Hip: *iliopsoas*	Stance	Increased hip flexion
	*adductors*	Throughout gait cycle	Adducted, scissoring gait
	*gluteus medius*	Throughout gait cycle	Internally rotated hip and foot progression angle

**Spasticity**	Ankle: *gastrocnemius, soleus*	Stance, Terminal swing	Increased ankle plantar flexion
	*posterior tibialis*	Throughout gait cycle	Ankle equinovarus
	Knee: *hamstrings*	Single limb support	Increased knee flexion
	Hip: *iliopsoas*	Pre-swing	Reduced hip extension
	*rectus femoris*	Terminal stance, Pre-Initial swing	Reduced hip extension, Reduced knee flexion in swing
	*adductors*	Throughout gait cycle	Adducted, scissoring gait
	*gluteus medius*	Throughout gait cycle	Internally rotated hip and foot progression angle

**Impaired SMC**	Ankle: *plantar flexor coupling with knee extensors*	IC, Terminal swing	Forefoot IC
	Ankle: *plantar flexor coupling with hip and knee extensors*	Midstance	Plantar flexed equinus gait → knee hyperextension in stance
	Knee: *knee flexor coupling with hip flexors*	Terminal swing	Flexed knee at IC
	Hip: *slow transition from terminal stance hip and knee extension to hip and knee flexion in initial swing*	Terminal stance – Midswing	Reduced hip and knee flexion in early swing → reduced foot clearance

*IC, initial contact*.

Flexed-knee gait can arise from short and spastic hip and knee flexors, as well as from weak hip extensors and ankle plantar flexors. Impaired SMC also contributes to flexed-knee gait and results in diminished knee extension during the terminal-swing phase of gait (Rha et al., 2016) (**Figure 3** and **Table 2**). Flexed-knee gait causes abnormal mechanical loads and muscle forces across the hip and knee during stance. In young children, that can result in bone deformities and a permanently flexed, rotated gait later in life (Bell et al., 2002; Steele et al., 2012). In the absence of full hip extension during early gait, the immature 30 degree femoral neck anteversion does not reduce to the normal 15 degrees of anteversion seen in TD children, resulting in internal hip rotation in children with CP (Shefelbine and Carter, 2004). Further compounding the problem, the internal rotation moment arms of hip muscles, such as the anterior gluteus medius, increase with flexed knee gait (Delp et al., 1999).

Stiff-knee gait arises from spastic knee extensors which impair pre-swing mechanics that limit knee flexion in early swing. In addition, hip flexor weakness impairs hip and knee flexion in early swing, and impaired SMC slows the transition from the stance phase hip and knee extension to the swing phase hip and knee flexion. The neuromuscular deficits contributing to stiff-knee gait primarily impact pre-swing through mid-swing phases of gait in spastic CP (**Figure 3** and **Table 2**). Here, we explore the influence of neuromuscular deficits on gait in the three types of CP with a focus on spastic CP.

#### The Influence of Weakness on Gait in Spastic CP: Treatment Considerations

The impact of weakness in spastic CP most notably affects ankle dorsiflexion in swing phase and hip and knee extension in stance phase. Thus, weakness affects all phases of the gait cycle (**Figure 3** and **Table 2**). Weak ankle dorsiflexors result in excessive ankle plantar flexion in swing and poor foot clearance with compensatory motion such as hip circumduction to clear the foot. Weak ankle dorsiflexors also cause excessive plantar flexion at initial contact, disrupting the normal heel-toe progression of gait. Weak plantar flexors fail to restrain tibial forward rotation over the foot in mid stance, increasing hip and knee flexion in stance. Furthermore, failure to stabilize the ankle during heel rise reduces the normal ankle plantar-flexion moment in terminal stance and can decrease peak knee flexion in swing. Weak hip and knee extensors contribute to flexed hip and knee postures during the stance phase of gait. Weak hip flexors can contribute to reduced peak knee flexion in swing. Weak hip abductors cause contralateral pelvic drop and increase ipsilateral trunk sway, shifting the body’s center of mass closer to the hips’ axis of rotation and thereby reducing the muscular demand on the hip abductors.

Steele et al. (2012) found children walking in flexed hip and knee gait had less passive skeletal support of body weight and utilize substantially higher muscle forces to walk than unimpaired individuals. Flexed hip and knee gait relied on the same muscles as unimpaired gait to accelerate the mass center upward, including the soleus, vasti, gastrocnemius, gluteus medius, rectus femoris, and gluteus maximus. However, these muscles were active throughout single-limb stance during flexed-knee gait in order to resist gravity, in contrast to the modulation of muscle forces seen during single-limb stance of unimpaired gait (Steele et al., 2012). Further, muscle weakness has been shown to have a negative effect on the Gait Profile Score, a measure of overall gait function (Schweizer et al., 2014).

Treatment with strength training targeting weak muscles has been found to be effective at improving gait, although further studies of targeted intensive strength training are needed. Evidence is accumulating around strength training as a means of improving mobility in CP without adverse effects (Dodd et al., 2002; Scholtes et al., 2012). To elucidate interventions that improve and maintain strength, i.e., force-generating capacity, and endurance, Fowler et al. (2010) studied 62 ambulatory children with spastic diplegic CP who underwent 30 intensive stationary cycling episodes over 12 weeks. They saw improvements in locomotor endurance, gross motor function, and some measures of strength in the cycling group but not the control group (Fowler et al., 2010). A review by Steele et al. (2012) found that individuals without hamstring spasticity had greater improvement in knee extension after strength training. In a study conducted by Scholtes et al. (2012), there was an improvement in muscle strength, but not mobility or spasticity, directly after training among 51 children with spastic CP who received 12 weeks of functional training; however, a detraining effect was observed 6 weeks after training ended. Jung et al. (2013) found that ankle plantarflexor strengthening improved strength and spatiotemporal gait parameters of six children with spastic CP. These subjects performed a heel raise exercise, which included progressive resistance ankle plantar flexor training for 6 weeks. In patients for whom weakness is a major contributor to gait deficits, strength training (as measured by force-generating capacity) in the extensor muscle groups may improve walking function and alignment, though further studies are needed to confirm kinematic and functional improvements (Damiano et al., 2010). Repetitive functional electrical stimulation to induce muscle strengthening has shown evidence of use-dependent muscle growth in children with CP with foot drop, though lasting improvements in voluntary ankle control have not been demonstrated (Damiano et al., 2013). More research is needed to develop the most effective treatment strategies for lasting improvements in strength that translate to improved gait function.

#### The Influence of Short Muscle-Tendon Units on Gait in Spastic CP: Treatment Considerations

Short muscle-tendon units of the hip and knee flexors and ankle plantar-flexors, particularly the bi-articular muscles, contribute to joint contracture and abnormal joint mechanics, and represent a primary cause of gait abnormalities in spastic CP (**Figure 3** and **Table 2**). Short plantar flexor muscle-tendon unit contributes to excessive ankle plantarflexion in stance and swing, leading to toe-walking or ‘equinus’ gait which limits foot clearance in swing. Equinus may co-exist with ankle dorsiflexor weakness, further impairing normal ankle dorsiflexion in swing phase. Limited foot clearance in swing causes compensatory movements such as hip circumduction in swing to clear the foot. A shortened posterior tibialis can contribute to equinovarus gait, which includes plantarflexion, inversion, and adduction of the foot. In cases of moderate to severe spastic CP where there is both distal and proximal limb involvement, flexed-knee gait is a common debilitating gait abnormality that creates abnormal mechanics across the hip, knee, and ankle that can result in bone deformity and increased fatigue while walking. Short semitendinosus contributes to two-thirds of all cases of flexed-knee gait (Arnold et al., 2006) at both initial contact and during the single limb support phase of the gait cycle. Musculoskeletal models of hamstring length and lengthening velocity just prior to initial contact quantify the contribution of short hamstrings to flexed knee gait (Arnold et al., 2006) and guide treatment decisions. Rha et al. (2016) found that a short medial gastrocnemius correlated with increased knee flexion at initial contact, whereas a short semitendinosus correlated with increased knee flexion at both initial contact and single limb support. In more severe cases, proximal involvement of hip flexors, adductors, and internal rotators contribute to flexed, adducted, and internally rotated gait. Recently, Kalsi et al. (2016) found an increased lengthening of the medial gastrocnemius during stance phase of gait in children with unilateral spastic CP compared to TD subjects, suggesting greater muscle excursion demands during gait.

Treatments for shortened muscle-tendon units in spastic CP include tendon lengthening and serial casting, however, both contribute to muscle weakness. In contrast, treatments that promote strength and increase muscle fiber diameter may also increase muscle length given the pennate angle of muscle fascicles (Shortland et al., 2001; Gough and Shortland, 2012). Treatment with muscle growth factors has not been well studied to date, however, it has potential to increase muscle size and length and warrants investigation as a treatment for CP. Work by Smith et al. (2009) and Barber et al. (2016) point to the importance of characterizing the nature of shortened muscle-tendon units and investigating growth factors as a potential therapy. While the impact of shortened muscle on gait has been fairly well studied, an optimal solution that preserves strength has not been developed. Further research can elucidate the links between WM brain injury, loss of normal neuromuscular activation to affected muscles, impaired musculoskeletal growth, short muscle-tendon unit, and the resulting gait abnormalities may lead to more effective treatments.

#### The Influence of Spasticity on Gait in Spastic CP: Treatment Considerations

Spasticity contributes to gait abnormalities by further compounding abnormal joint postures and by restraining normal rapid flexion or extension during gait. Spasticity contributes to excessive ankle plantarflexion, knee flexion, and hip flexion, adduction, and/or rotation (**Figure 3** and **Table 2**). Plantarflexor spasticity increases the equinus deformity of the ankle contributing to toe-walking and forefoot only contact in stance and poor foot clearance in swing. Spasticity of the posterior tibialis increases the equinovarus deformity of the foot, which includes ankle plantarflexion, inversion, and adduction. Hamstrings spasticity can limit knee extension in terminal swing and lead to increased knee flexion at initial contact. Indeed, Steele et al. (2012) found that hamstring spasticity was associated with an undesirable increase in knee flexion during walking. Stiff-knee gait can result from increased spasticity and passive resistance to stretch in the rectus femoris, thereby restricting the normal rapid knee flexion in early swing and limiting peak knee flexion. Hip adductor spasticity can lead to an adducted, scissoring gait, contributing to tripping. Spasticity of the hip internal or external hip rotators will contribute to an internally or externally rotated hip, respectively.

Treatments for spasticity include BoNT-A injections, oral or intrathecal baclofen, and dorsal rhizotomy. However, treatments that promote strength may also be effective. A study conducted by Williams et al. (2013) found that simultaneous use of BoNT-A and strength training was successful in spasticity reduction, improving strength, and achieving functional goals, over and above treatment with BoNT-A alone in 15 children (aged 5–12 years) with spastic diplegic CP. Further research is needed to assess the efficacy of treatments that promote strength, such as intensive strength training and functional electrical stimulation, for reducing spasticity and improving overall gait function.

#### The Influence of Impaired Selective Motor Control on Gait in Spastic CP: Treatment Considerations

Impaired SMC results in obligatory muscle co-activation of flexor or extensor muscle synergies during gait. For example, at least three gait events are impacted by impaired SMC (**Figure 3** and **Table 2**). Ankle plantarflexion in terminal swing may increase when the knee is extending as part of an extensor synergy, leading to a forefoot initial contact. Increased ankle plantarflexion may also occur in stance when the knee is normally extended as part of an extensor synergy, leading to equinus gait and knee hyperextension. Knee extension in terminal swing may be limited when the hip is normally flexed as part of a flexor synergy, leading to a flexed-knee posture at initial contact. Rha et al. (2016) showed that while a short semitendinosus correlated with increased knee flexion at both initial contact and single limb support, impaired SMC assessed using the SCALE proved to be a stronger correlate of knee flexion at initial contact than semitendinosus length. In addition, a slow transition from hip and knee extension in terminal stance to hip and knee flexion in initial swing leads reduced foot clearance in swing due to reduced hip and knee flexion. Chruscikowski et al. (2017) studied 194 patients with bilateral CP and found a significant, negative correlation between SMC measured using SCALE and gait impairment, as measured by Gait Profile Score, suggesting that impaired SMC negatively affects gait function.

Recently, Shuman et al. (2016) examined EMG patterns of muscle activation during gait in CP and found increased patterns of muscle synergies. They developed an EMG analysis to quantify synergy patterns during gait and found that prolonged antigravity muscle activation is necessary to prevent collapse in flexed-knee gait. However, prolonged and simultaneous antigravity muscle activation may be a compensatory symptom, separate from impaired SMC and therefore requires further delineation.

Treatments utilizing intensive exercise patterns outside of synergy patterns, such as knee extension combined with ankle dorsiflexion, may prove beneficial and warrant further investigation. Research is needed to determine the efficacy of intensive exercise on improving SMC using exercise patterns that combine flexion and extension of adjacent joints. Further work is needed to determine if improvements in SMC translates to improved gait patterns.

### The Influence of Neuromuscular Deficits on Gait in Dyskinetic CP

Dyskinetic CP is characterized by involuntary hyperkinetic or repetitive dystonic limb movements that impair motor function (Sanger, 2006). Primitive reflexes are more prominent and persist for a longer time in dyskinetic CP and may interfere with gait (Sankar and Mundkur, 2005). The influence of neuromuscular deficits in dyskinetic CP is not well studied. However, the increased movement variability and involuntary muscle contractions in children with dyskinetic CP (Sanger, 2006; Sanger et al., 2010) undoubtedly impact gait. Further, children with dystonia are at risk of developing fixed musculoskeletal deformities, which progress faster with worsening GMFCS level and among children with both dystonia and spasticity (Lumsden et al., 2016). Determining whether dyskinetic movements are random and variable or involve a small number of specific abnormal motor patterns (Sanger, 2006) will clarify the influence on gait and guide more effective treatment.

Deep brain stimulation to the globus pallidus internus may be an effective treatment option for children with dyskinetic CP (Review: Koy et al., 2013). It has been shown that response to pallidal deep brain stimulation in the treatment of dystonia yields better outcomes if administered earlier in life (<7 years of age) (Lumsden et al., 2013), highlighting a need for early intervention.

### The Influence of Neuromuscular Deficits on Gait in Ataxic CP

Ataxic CP is characterized by impaired limb coordination, balance, and stability (Hughes and Newton, 1992; Schnekenberg et al., 2015; Graham et al., 2016). These neuromuscular deficits impose instability and result in a compensatory wider base of support and elevated, outreaching arm postures to improve balance during gait. In addition, a more circuitous or less direct gait path may be observed. Little work has been done to specifically characterize the neuromuscular deficits in ataxic CP and their influence on gait. Better characterization of the deficits can help develop more effective treatment methods.

### Neural Correlates of Gait Abnormalities in CP

To date, few studies have examined the neural correlates of motor deficits and gait abnormalities. Staudt et al. (2003) found correlations (*r* = 0.8; *p* < 0.01) between lower limb motor dysfunction assessed with the Movement ABC and extent of contralateral PVL, as assessed on semi-coronal reconstructions from 3D-MRI in 13 adolescents with CP. In addition, Rademaker et al. (2004) found an inverse association between motor function assessed on Movement ABC scores and corpus callosum area assessed on MRI in 204 preterm children with VLBW. The association existed in frontal, middle, and posterior corpus callosum areas but increased in the direction of the posterior part. Lee et al. (2011) found a strong relationship between motor dysfunction assessed with GMFCS and fractional anisotropy values assessed on DTI within the bilateral CST and posterior body of the corpus callosum (*p* < 0.03); Cortical volume of the pre- and post-central gyri, and the paracentral lobule was negatively associated with GMFCS levels (*p* < 0.005).

Meyns et al. (2016b) found that the severity of gait abnormality assessed using the Gait Profile Score correlated to total corpus callosum (*r* = -0.441; *p* < 0.040) and subpart 1 (*r* = -0.437; *p* < 0.042) volumes in children with CP (Witelson, 1989). Furthermore, Rose et al. (2007) found evidence to support early prognosis of gait abnormalities in VLBW preterm children. They found that neonatal WM microstructure of the posterior limbs of the internal capsule assessed with DTI fractional anisotropy correlated to severity of gait abnormalities at 4 years of age, measured using 3D kinematics (*r* = 0.89, *p* < 0.0) and to GMFCS (*r* = 0.65, *p* < 0.04). In a separate cohort of VLBW preterm children, gait velocity at 18–22 months of age correlated (*r* = -0.374, *p* < 0.007) with near term brain microstructure in the genu of the corpus callosum assessed on DTI mean diffusivity (Rose and McGill, 1998).

## Conclusion

Emerging evidence suggest that important links exist between neurologic injury, neuromuscular deficits, and specific gait abnormalities in CP. A better understanding of these relationships can elucidate underlying mechanisms of gait impairments and lead to more strategic treatments. Here, we have reviewed the nature of brain injuries in CP, the associated neuromuscular deficits, and the subsequent gait abnormalities on both a microscopic and functional level.

Our understanding of brain abnormalities in CP have been informed by rapidly evolving neuroimaging techniques. However, inconsistent methodology and reporting of data limit interpretation. These discrepancies have been identified as “(1) inappropriate assignment of etiology to morphologic findings, (2) inconsistent descriptions of radiologic findings, (3) uncertain relationship of pathologic findings to brain insult timing estimates, and (4) study designs that are not based on generalizable samples” (Korzeniewski et al., 2008). In addition, consistent, standardized language across studies would help compare findings in context of the existing body of knowledge. Neuroimaging findings are rarely reported with anatomical descriptions sufficient for direct comparison between studies. For example, the localization of atrophy of GM or WM, the location of cavities, and the nature of anomalies found on imaging are not specified in a consistent manner. However, the National Institute of Neurological Disorders and Stroke (NINDS), in conjunction with the American Academy for Cerebral Palsy and Developmental Medicine (AACPDM) have recently developed a set of Common Data Elements for use in CP research, including neuroimaging diagnostics, which promises to standardize clinical language and improve data collection consistency for future research on CP (Odenkirchen et al., 2016).

The criteria for estimating the time of injury are also not well described; thus, exactly how prenatal and perinatal insults are differentiated is unclear, and there is a need to develop accurate, standardized techniques. Automated analysis of MRI is an exciting new field that can facilitate precise identification of brain abnormalities and improve consistency in the interpretation of MRI scans. Automated brain lesion segmentation using the Least Absolute Shrinkage and Selection Operator (LASSO) on both WM and GM from T1-weighted MRI sequences was recently validated against manual expert classifications of lesions (Pagnozzi et al., 2016). The prognostic ability of MRI to determine mild motor impairment and exactly which motor functions will be compromised is still limited. Thus, further work on developing a quantitative relationship between lesion burden and functional outcome will help increase the utility of structural MRI in predicting individual prognoses and planning targeted therapeutic interventions.

Precision in identifying microstructural brain abnormalities on DTI will also advance our understanding of brain abnormalities in CP. Current DTI techniques are limited by spatial resolution, intravoxel averaging of anisotropy by adjacent tracts, partial volume effects, and image artifacts. DTI imaging of the reticulospinal and rubrospinal tracts will improve our understanding of the contribution of these tracts to gait abnormalities in children with CP. Developments to the diffusion tensor model, which uses optimized acquisition schemes, such as high angular resolution diffusion imaging (HARDI) (Tuch et al., 2002) and higher-order modeling of diffusion anisotropy, will improve the resolution of crossing fibers within each voxel. With better resolution, more accurate estimations of tract injury or plasticity within corticomotor networks can be made (Rose et al., 2011). Multi-modal imaging such as functional neuroimaging (functional MRI or magnetoencephalography) guided DTI holds promise for correlating structure-function relations in children with CP (Reid et al., 2016). Improved techniques to precisely quantify neuromuscular deficits in CP are also needed to clarify these important structure-function relations.

By discussing the neurologic correlates of gait abnormalities within this context, we hope to encourage the reader to recognize specific mechanisms of gait abnormalities in CP and discover targeted treatment opportunities that can substantially improve functional outcomes for children with CP.

## Author Contributions

JZ, EB, and JR synthesized literature and wrote the paper. All authors discussed and commented on the manuscript.

## Conflict of Interest Statement

The authors declare that the research was conducted in the absence of any commercial or financial relationships that could be construed as a potential conflict of interest.

## Abbreviations

ADC: apparent diffusion coefficient
ATP: adenosine triphosphate
BGTL: basal ganglia and/or thalamus lesions
BoNT-A: botulinum toxin-A
CP: cerebral palsy
CST: corticospinal tract
CT: computed tomography
DTI: diffusion tensor imaging
EMG: electromyography
GM: gray matter
GMFCS: Gross Motor Function Classification Scale
MRI: magnetic resonance imaging
MVC: maximal voluntary contraction
PVL: periventricular leukomalacia
SCALE: Selective Control Assessment of the Lower Extremity
SMC: selective motor control
TD: typically developing
VLBW: very low birthweight
WM: white matter
